# *In vitro* transdifferentiated signatures of goat preadipocytes into mammary epithelial cells revealed by DNA methylation and transcriptome profiling

**DOI:** 10.1016/j.jbc.2022.102604

**Published:** 2022-10-17

**Authors:** Xiao-Ru Yan, Tao Shi, Jia-Ying Xiao, Ya-Fang Liu, Hui-Ling Zheng

**Affiliations:** College of Animal Science and Technology, Northwest A&F University, Yangling, Shaanxi, China

**Keywords:** preadipocyte, transdifferentiation, mammary epithelial cell, transcriptome, DNA methylation, ACTG1, actin gamma 1, ALB, albumin, ASC, adipose-derived stem cell, BACH2, BTB domain and CNC homolog 2, cDNA, complementary DNA, CM, conditioned media, CTCF, CCCTC-binding factor, DEG, differentially expressed gene, DLK1, delta-like noncanonical Notch ligand 1, DMEM, Dulbecco's modified Eagle's medium, DMG, differentially methylated gene, DML, differentially methylated location, DMR, differentially methylated region, ECM, extracellular matrix, FBS, fetal bovine serum, GMEC, goat MEC, GO, Gene Ontology, GM-preadipocyte, goat mammary preadipocyte, HSP90AA1, heat shock protein 90 alpha family class A member 1, KEGG, Kyoto Encyclopedia of Genes and Genomes, KRT18, keratin 18, MEC, mammary epithelial cell, NG, gene that has significantly negative correlation, NID1, nidogen 1, PDGFRA+, platelet-derived growth factor receptor alpha (+), *PLCL1*, phospholipase C like 1, PPAR, peroxisome proliferator–activated receptor, RND1, Rho family GTPase 1, RT–qPCR, real-time quantitative PCR, *SPPL3*, signal peptide peptidase like 3, STAT, signal transducer and activator of transcription, TEM, transmission electron microscope, TF, transcription factor, WGBS, whole genome bisulfite sequencing

## Abstract

During mammary development, the transdifferentiation of mammary preadipocytes is one of the important sources for lactating mammary epithelial cells (MECs). However, there is limited knowledge about the mechanisms of dynamic regulation of transcriptome and genome-wide DNA methylation in the preadipocyte transdifferentiation process. Here, to gain more insight into these mechanisms, preadipocytes were isolated from adipose tissues from around the goat mammary gland (GM-preadipocytes). The GM-preadipocytes were cultured on Matrigel in conditioned media made from goat MECs to induce GM-preadipocyte-to-MEC transdifferentiation. The transdifferentiated GM-preadipocytes showed high abundance of keratin 18, which is a marker protein of MECs, and formed mammary acinar-like structures after 8 days of induction. Then, we performed transcriptome and DNA methylome profiling of the GM-preadipocytes and transdifferentiated GM-preadipocytes, respectively, and the differentially expressed genes and differentially methylated genes that play underlying roles in the process of transdifferentiation were obtained. Subsequently, we identified the candidate transcription factors in regulating the GM-preadipocyte-to-MEC transdifferentiation by transcription factor–binding motif enrichment analysis of differentially expressed genes and differentially methylated genes. Meanwhile, the secretory proteome of GM-preadipocytes cultured in conditioned media was also detected. By integrating the transcriptome, DNA methylome, and proteome, three candidate genes, four proteins, and several epigenetic regulatory axes were further identified, which are involved in regulation of the cell cycle, cell polarity establishment, cell adhesion, cell reprogramming, and adipocyte plasticity. These findings provide novel insights into the molecular mechanism of preadipocyte transdifferentiation and mammary development.

The mammary gland is a tissue that develops throughout the sexual maturity and performs physiological function of milk synthesis and secretion. It is mostly made up of epithelial tissue and stroma, which contains adipose tissue known as the “fat pad.” The fat pad in mammary gland is required for mammary development *via* signals that trigger the ductal morphogenesis (1). There are three kinds of adipocytes in fat pad, white adipocytes that store and breakdown lipids, brown adipocytes that are thermogenic, and pink adipocytes with lactation function (2). Under certain circumstances, all three types of adipocytes have been shown to coexpress *leptin* and *perilipin A* and transform accordingly (3). When hormone levels fluctuate during different stages of development, they may also go through a series of modifications in the mammary gland (4). Furthermore, adipocytes remain active throughout mammary development, demonstrating a significant amount of flexibility while transitioning from adipocytes to fibroblast-like cells and preadipocytes. The mRNA and protein levels of delta-like noncanonical Notch ligand 1 (DLK1) are highly expressed in preadipocytes and absent in mature adipocytes (5, 6, 7). The mRNA and protein levels of CD34 molecule are highly expressed in preadipocytes and lowly expressed in mature adipocytes (8, 9). Hence, DLK1 and CD34 have been utilized as marker proteins of preadipocytes. The human preadipocytes marked with DLK1 cultured in the medium made from human mammary epithelial cells (MECs) in 3D culture condition transdifferentiated into adult MECs (5). Preadipocytes tagged with platelet-derived growth factor receptor alpha (+) (PDGFRA+), as a source of epithelial descendents, depleted during mammary acini production after transdifferentiating into MECs (also known as “pink adipocytes”) (5, 6, 7, 10). Keratin 18 (KRT18) is highly expressed in monolayer or simple epithelial tissue and was used as a marker protein of MECs (11, 12).

Acinus is a spherical body of monolayer MECs that surrounds center lumen, and these acini in the mammary gland are involved in lactation. The production of acinus occurs in two ways: (i) the proliferation and differentiation of mammary epithelial stem cells, which are associated with expansion and maturation of the epithelial portion and (ii) pregnancy-induced transdifferentiation of mammary adipocytes to MECs (13). MECs are derived from acini progenitor cells in the first stage of pregnancy, and lipid-laden MECs develop in the second stage when subcutaneous adipocytes shrink and exhibit epithelial-like characteristics to transdifferentiate into MECs. However, these cells produce the secretory acini with myoepithelial cells (3, 14) that mature in three consecutive steps. The first is proliferation, in which single cells grow into multicellular clusters; the second is secession, in which outer layer of cells establishes a polarity axis and undergoes proliferative suppression; and the third is apoptosis, in which inner cells die, resulting in the formation of hollow lumen (15, 16). The transdifferentiated preadipocytes also undergo the process of acinar formation. We have known that preadipocyte-to-MEC transdifferentiation is crucial for mammary development because it also involves in the production of acinar structure. However, earlier work has revealed little about the processes of dynamic control of transcriptome and genome-wide DNA methylation in preadipocyte transdifferentiation, and there has been little in-depth investigation into the molecular mechanisms involved in preadipocyte transdifferentiation.

Although mouse models of mammary development are very valuable and easy to handle, they have inherent limitations, and caution is recommended while applying the results of development studies directly to humans (17). Previous research has indicated that ruminant mammary could be utilized as a good supplementary model for the study of mammary development because of in-depth understanding development process and microanatomical of ruminant mammary (18). Therefore, in this study, the preadipocytes were isolated from adipose tissues from around the goat mammary gland (GM-preadipocytes), and the cultured medium of goat MECs (GMECs) grown on Matrigel was collected as the conditioned media (CM) to induce GM-preadipocyte-to-MEC transdifferentiation. Furthermore, GM-preadipocytes and transdifferentiated GM-preadipocytes performed transcriptome and DNA methylome sequencing to screen differentially expressed genes (DEGs) and differentially methylated genes (DMGs). Moreover, the proteome of CM was also detected. The candidate genes, proteins, and epigenetic regulatory networks involved in GM-preadipocyte-to-MEC transdifferentiation were further identified through integration analysis of the transcriptome, DNA methylome, and the proteome. This work will provide novel insights into molecular mechanism of adipocyte plasticity and supply a theoretical underpinning for the mammary development.

## Results

### The characterization of GMECs and GM-preadipocytes

The GM-preadipocytes were distinguished from GMECs by Oil Red O staining and immunofluorescence of marker proteins. Preadipocyte marker proteins included DLK1 and CD34, whereas epithelial cell marker protein included KRT18. During adipogenic induction, the growth medium of GM-preadipocytes was changed to adipogenic induction medium at day 0. A small number of lipid droplets in cells were observed at day 2. The GM-preadipocytes have differentiation potential, as indicated by the increase in lipid droplet formation at day 5 and the remarkable increase in droplet size that continued until day 8 (Fig. S1*A*). Moreover, CD34 and DLK1 were highly expressed in GM-preadipocytes, and KRT18 was found to be expressed strongly in GMECs (Fig. S1*B*). These findings showed that GM-preadipocytes and GMECs may be utilized for future researches.

### Morphological changes of the GMECs cultured in Matrigel

**Figure 1:**
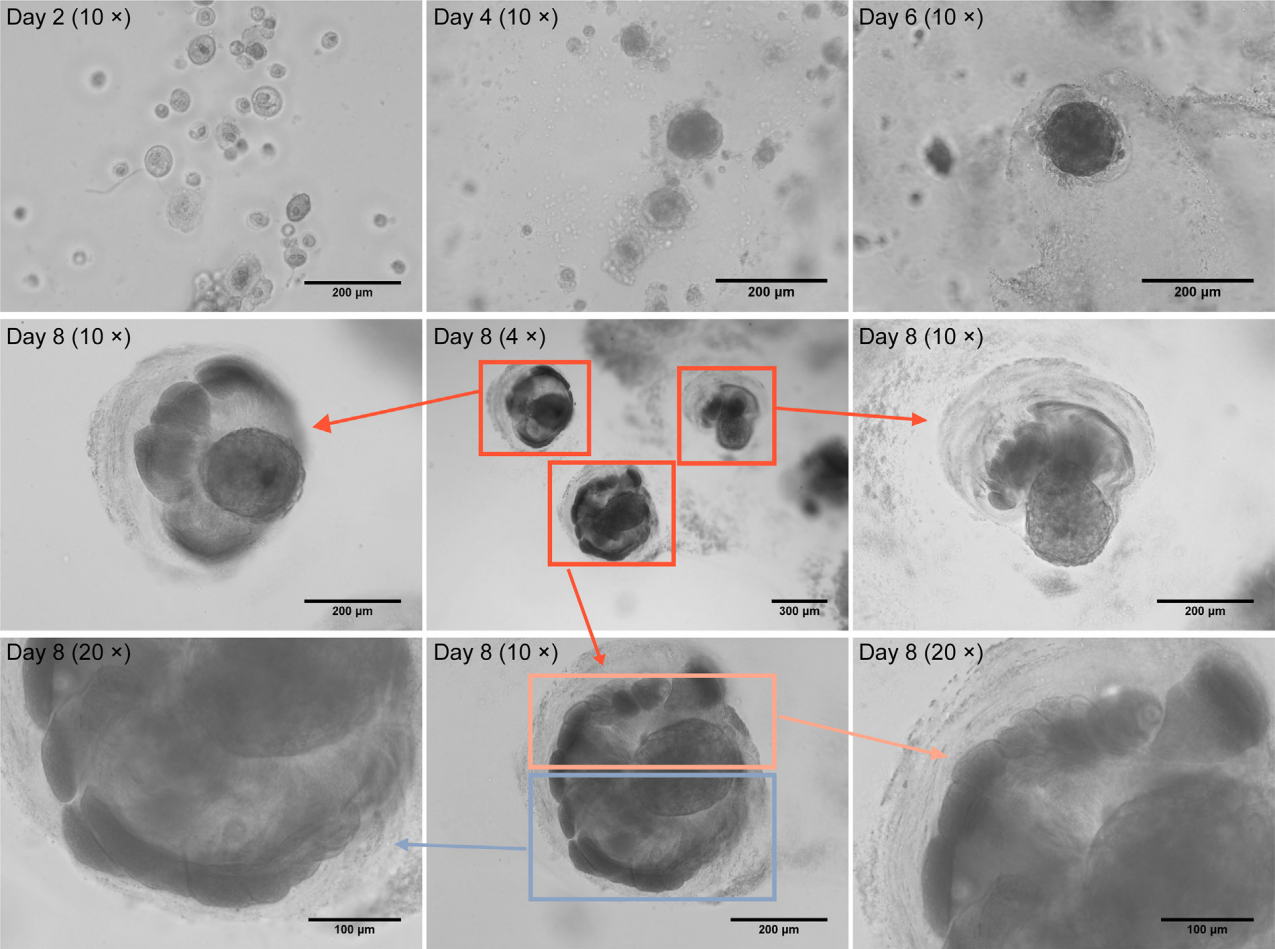
**The morphological characteristics of the epithelial structures formed by GMECs cultured in Matrigel.** The morphological changes of GMECs at day 2, 4, 6, and 8 of spontaneous induction, and the cell suspension was inoculated on Matrigel at day 0. Cells were embedded in Matrigel at day 2, and at day 4, cell clusters were gradually formed. At day 6, cell clusters got bigger, and at day 8, acinar structures and lumen appeared. The *red boxes* represent three acinar structures, respectively (magnification: 4×), and the *red arrows* show magnifications of the separate acinar structure (magnification: 10×). The *orange* and *purple boxes* represent different parts of an acinar structure, respectively (magnification: 10×), and the *arrows* with corresponding colors show magnifications of the *upper* and *lower parts* of the acinar structure (magnification: 20×), magnification in *brackets*. GMEC, goat mammary epithelial cell.

GMECs were cultured with the growth medium in Matrigel to simulate the conditions seen *in vivo* to determine whether they might spontaneously induce to the formation of acinar structures. During spontaneous induction of GMECs, the cell suspension was inoculated on Matrigel at day 0. Then we observed cells regularly and stably embedded in Matrigel at day 2; and cell clusters gradually formed at day 4, suggesting the establishment of polarity of outer cells and reduction of proliferative activity; at day 6, cell clusters got bigger, demonstrating the presence of cell adhesion; and at day 8, acinar structures and lumen appeared, indicating that the inner cells suffered apoptosis (Fig. 1). These findings showed that the GMECs cultured on Matrigel-coated dishes produced acinar structures and lumens.

### Different behaviors of the GM-preadipocytes induced by the CM

**Figure 2:**
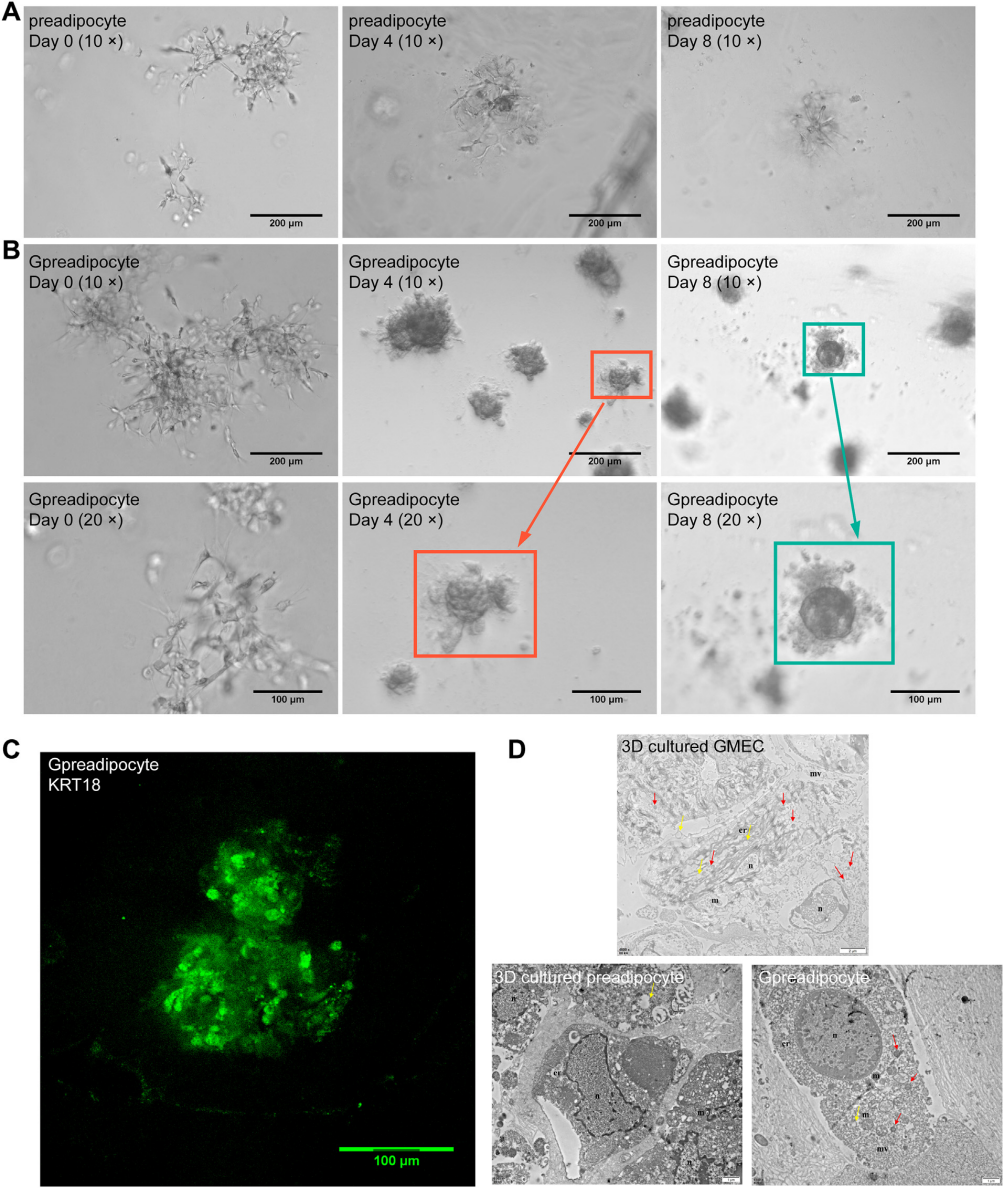
**Different behaviors of GM-preadipocyte induced by the CM.***A*, morphological changes of GM-preadipocyte in the Preadipocyte group (magnification: 10×). *B*, morphological changes of GM-preadipocyte in the Gpreadipocyte group. The cells were reticular at day 0; the antennae of cells shrank at day 4; the cells were developed in a swirling pattern to form colonies, acinar structure, and lumen at day 8 (magnification: 10×). The *red* and *green boxes* represent the cell clusters in the process of GM-preadipocyte transdifferentiation, respectively (magnification: 10×), and the *arrows* with corresponding colors show magnifications of the indicated *red* and *green rectangular* region (magnification: 20×), magnification in *brackets*. Day 0 refers to the day when GM-preadipocytes undergone serum starvation and were treated with CM. *C*, immunofluorescence of Gpreadipocyte group using KRT18 monoclonal antibody (*green*) under confocal laser scanning microscope. *D*, the subcellular structures of the GMEC, Preadipocyte, and Gpreadipocyte groups. The *yellow* and *red arrows* represent desmosome joining adjacent cells and dense apical granules, respectively. CM, conditioned media; er, endoplasmic reticulum; GMEC, goat mammary epithelial cell; GM-preadipocyte, goat mammary preadipocyte; KRT18, keratin 18; m, mitochondrion; mv, microvilli; n, nucleus.

The CM were utilized to induce GM-preadipocyte-to-MEC transdifferentiation in Matrigel to determine the effect of secretory functional factors from GMEC culture medium on GM-preadipocytes. The cell morphology was not significantly changed in Preadipocyte group (Fig. 2*A*). However, in the Gpreadipocyte group, when cells were induced with CM, cells were reticular at day 0; the antennae of cells shrank at day 4, indicating epithelialization of GM-preadipocytes. At day 8, cells formed colonies, acinar structures, and lumens in a swirling pattern, indicating that the GM-preadipocytes experienced polarity establishment, proliferative suppression, cell adhesion, and apoptosis of inner cells (Fig. 2*B*). Interestingly, the acinar structures generated from GM-preadipocytes in Gpreadipocyte group were comparable to those of GMECs.

Simultaneously, immunofluorescence results revealed that GM-preadipocytes transdifferentiated into MECs with high KRT18 expression (Fig. 2*C*). Transmission electron microscope (TEM) was used to observe if transdifferentiated GM-preadipocytes established a polarity axis and formed acinar structures. In the Gpreadipocyte group, there were many microvilli, acrosome particles, and desmosomes that connected adjacent cells in the cytoplasm, and a significant number of mitochondria, which were identical to GMEC organelles (Fig. 2*D*). These findings showed that CM enabled GM-preadipocytes to transdifferentiate into MECs, establish polarity axis, and form acinar structures.

### Transcriptome profiling of the GM-preadipocytes pre- and post-transdifferentiation

The transcriptome sequencing of GM-preadipocytes and transdifferentiated GM-preadipocytes was used to screen candidate genes that involved in the transdifferentiation. The sequencing results showed an average of 44,475,402 and 42,430,324 raw reads generated, respectively, whereas 42,691,545 and 41,727,560 clean reads were obtained accordingly after removing low-quality reads, with about 90.85% and 90.63% of clean reads independently mapped to goat reference genome ARS1 in the Preadipocyte and Gpreadipocyte groups, respectively (Table 1).

**Table 1:** The summary of data generated by transcriptome sequencing

Sample ID	Raw data (G)	Raw reads	Clean reads	Q30 (%)	Mapping rate (%)
Control 1	6.69	44,622,186	42,663,980	92.81	89.76
Control 2	6.21	41,431,868	39,394,432	93.22	91.06
Control 3	6.98	46,511,600	44,716,412	93.12	91.14
Control 4	6.66	44,409,918	43,179,252	93.31	91.23
Control 5	6.81	45,401,436	43,503,648	93.37	91.04
Treatment 1	6.61	44,034,266	43,399,386	93.26	89.94
Treatment 2	6.23	41,529,492	40,715,226	93.40	92.05
Treatment 3	5.98	39,863,256	39,005,840	93.04	89.50
Treatment 4	6.22	41,488,544	40,888,234	92.85	91.50
Treatment 5	6.79	45,236,064	44,629,116	92.42	90.14

Control: the Preadipocyte group; treatment: the Gpreadipocyte group.

**Figure 3:**
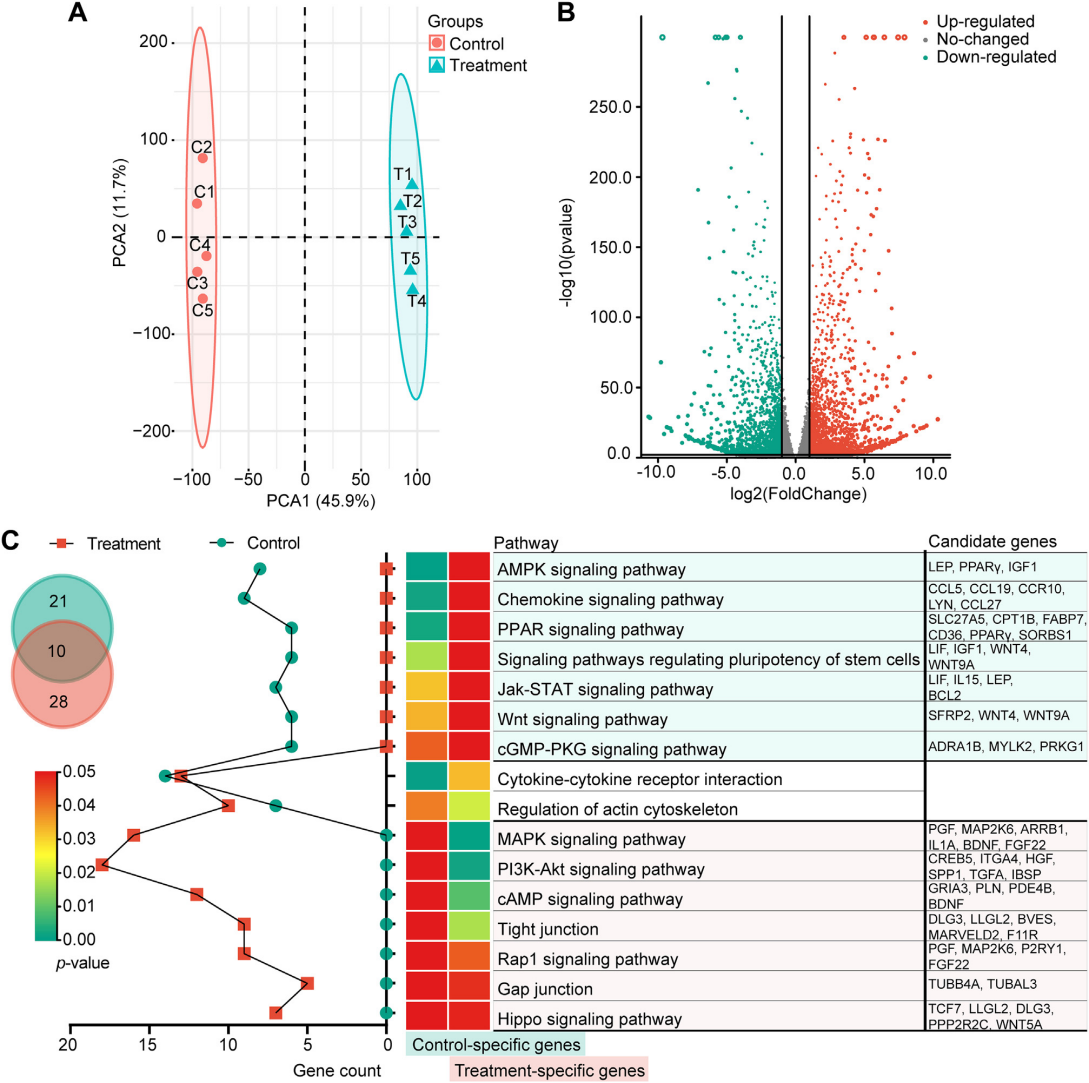
**Transcriptomic profiling of the GM-preadipocytes pre- and post-transdifferentiation.***A*, PCA of transcriptome. *B*, volcano plots of mRNA. *C*, KEGG pathway enrichment analysis of stage-specific genes. Venn diagram represents the intersection of pathways enriched by stage-specific genes in control and treatment groups. Control and treatment groups represent the Preadipocyte and Gpreadipocyte groups, respectively. GM-preadipocyte, goat mammary preadipocyte; KEGG, Kyoto Encyclopedia of Genes and Genomes; PCA, principal component analysis.

There were no outlier samples in either of two groups according to the principal component analysis (Fig. 3*A*). The *PDGFRA* expression found in the transcriptome of Preadipocyte group indicated that the GM-preadipocytes were *PDGFRA* (+) preadipocytes. The mRNA profiles of Preadipocyte and Gpreadipocyte groups indicated 823 and 887 stage-specific genes, respectively (Table S1). In comparison to the Preadipocyte group, the Gpreadipocyte group had 4337 DEGs, of which 2238 were upregulated, including several epithelial marker genes, such as factor V/VIII domain containing (*MFGE8*), perilipin 2 (*Plin2*), and E74 like ETS transcription factor (TF) 5 (*Elf5*); and 2099 were downregulated, including adipocyte marker genes like peroxisome proliferator–activated receptor gamma (*PPARγ*) and *FABP4* (Fig. 3*B* and Table S2). Subsequently, the 10 genes selected from all DEGs were verified *via* real-time quantitative PCR (RT–qPCR). The results were in accordance with the transcriptome data, indicating that transcriptome sequencing generated reliable data (Fig. S2).

Furthermore, the functions of stage-specific genes were investigated by Kyoto Encyclopedia of Genes and Genomes (KEGG) pathway enrichment analysis, and the stage-specific genes of the Preadipocyte group were enriched in PPAR, Jak- signal transducer and activator of transcription (STAT), Wnt signaling pathways, and the regulation of pluripotency of stem cell pathway. Whereas, the stage-specific genes of the Gpreadipocyte group were enriched in tight junction, gap junction, Rap1, MAPK, PI3K-Akt, and Hippo signaling pathways, which highlighted the central roles of the cell adhesion, morphogenesis, and organogenesis during the process of transdifferentiation (Fig. 3*C* and Table S3). Among them, both PPAR and Hippo signaling pathways have been proved to associate with the adipocyte transdifferentiation and acinar formation.

**Figure 4:**
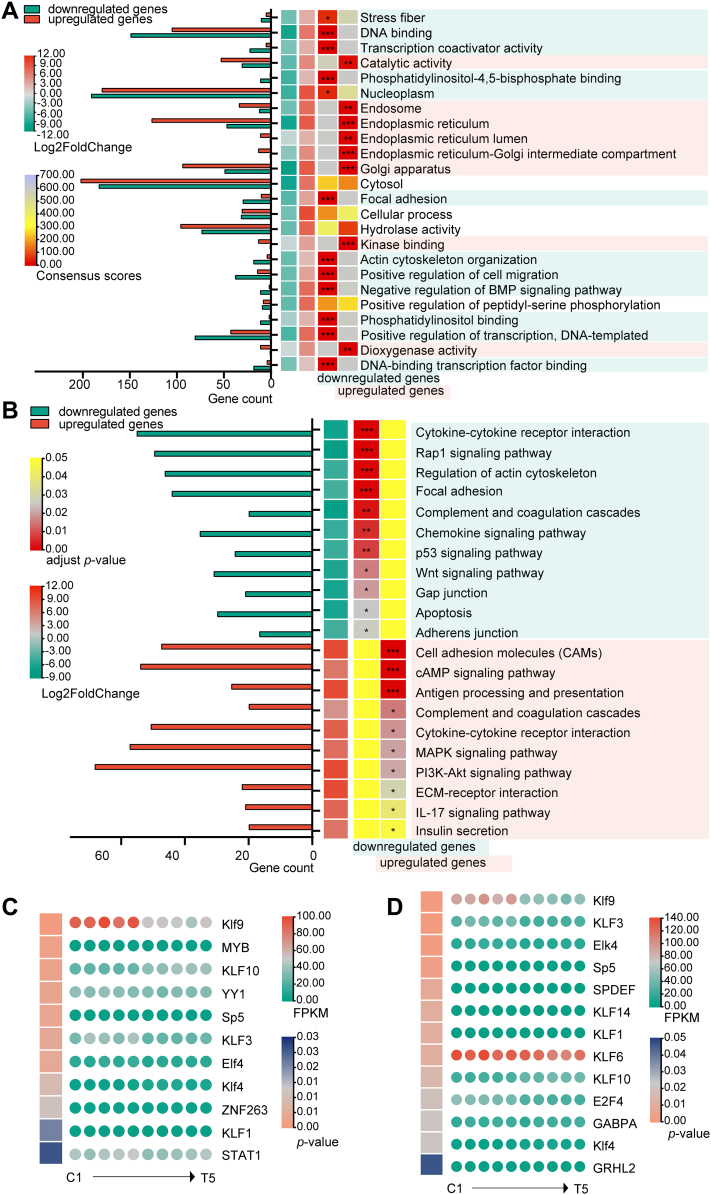
**Enrichment analysis of GSEA, KEGG, and TF motifs for DEGs.***A*, GSEA of DEGs. *B*, KEGG pathway enrichment analysis of DEGs. *C* and *D*, TF-binding motif enrichment analysis around the promoter of downregulated and upregulated DEGs, respectively. DEG, differentially expressed gene; GSEA, gene set enrichment analysis; KEGG, Kyoto Encyclopedia of Genes and Genomes; TF, transcription factor.

Simultaneously, gene set enrichment analysis was used to evaluate the potential function of DEGs, and results showed that downregulated and upregulated DEGs were enriched in different Gene Ontology (GO) terms. The downregulated DEGs were significantly enriched in GO terms including focal adhesion, actin cytoskeleton organization, DNA-binding TF binding, and transcription coactivator activity. Whereas upregulated DEGs were evidently enriched in GO terms including catalytic activity, endosome, and Golgi apparatus (Fig. 4*A* and Table S4). Moreover, results from KEGG pathway enrichment analysis showed that upregulated DEGs were significantly involved in cell cycle and cell adhesion, whereas downregulated DEGs were strongly associated with the immune response, cell cycle, cell adhesion, morphogenesis, and organogenesis (Fig. 4*B* and Table S5). The overall GO and KEGG enrichment analysis demonstrated that identified DEGs were closely related to regulation of cell cycle, cell adhesion, and morphogenesis.

The promoter motif enrichment analysis was used to determine if DEGs were collectively controlled by certain TFs. We found 11 and 13 binding motifs for downregulated and upregulated DEGs, respectively. These discovered TFs might play critical roles in the various processes of GM-preadipocyte-to-MEC transdifferentiation (Fig. 4, *C*, *D* and Table S6).

### DNA methylation patterns of the GM-preadipocytes pre- and post-transdifferentiation

The DNA methylation patterns in the GM-preadipocyte-to-MEC transdifferentiation were studied using whole genome bisulfite sequencing (WGBS). In the Preadipocyte and Gpreadipocyte groups, an average of 328,480,510 and 349,232,847 raw reads were generated, 227,236,312 and 225,855,304 clean reads were obtained, about 93.38% and 93.21% of clean reads were uniquely mapped to goat reference genome ARS1, respectively (Table 2).

**Table 2:** The summary of data generated by WGBS

Sample ID	Raw data (G)	Raw reads	Clean reads	Mapping rate (%)
Control 1	67.36	336,798,123	244,789,294	93.22
Control 2	61.26	306,348,087	184,612,121	93.42
Control 3	70.12	350,612,933	252,307,520	93.50
Treatment 1	62.42	312,090,750	198,212,072	93.22
Treatment 2	85.96	429,888,384	301,301,906	93.21
Treatment 3	61.14	305,719,408	178,051,934	93.21

Control: the Preadipocyte group; treatment: the Gpreadipocyte group.

**Figure 5:**
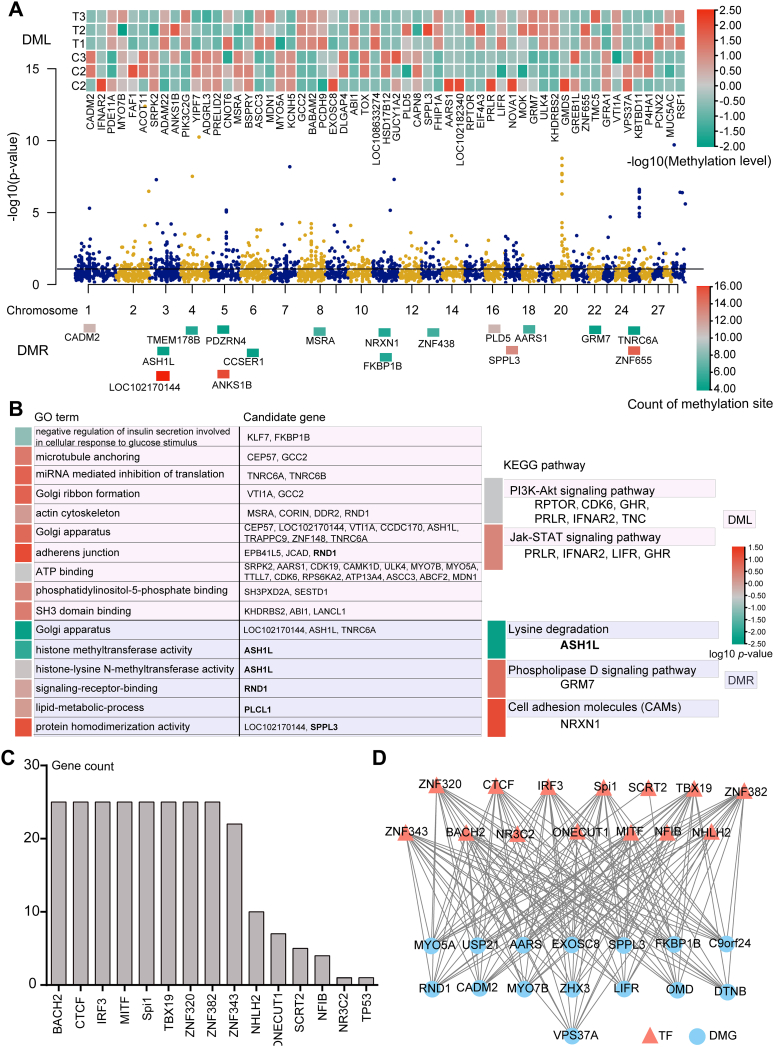
**DNA methylation profiling of the GM-preadipocytes pre- and post-transdifferentiation.***A*, Manhattan map of DMGs, including the methylation degree of DMLs and the number of methylation sites in DMRs on every chromosome. *B*, GO and KEGG enrichment analysis of DMGs. *C*, TF-binding motif enrichment analysis around the promoter of DMGs. *D*, the DNA methylation–meditated TF-gene regulatory network of GM-preadipocyte-to-MEC transdifferentiation. DMG, differentially methylated gene; DML, differentially methylated location; DMR, differentially methylated region; GM-preadipocyte, goat mammary preadipocyte; GO, Gene Ontology; KEGG, Kyoto Encyclopedia of Genes and Genomes; MEC, mammary epithelial cell; TF, transcription factor.

Besides, principal component analysis results revealed no outliers in all samples (Fig. S3). Moreover, the methylation degrees of different sites on each chromosome were obtained (Table S7). Average CpG counts were 7,639,657 and 5,039,938, and methylated CpG represented 63.97% and 62.83% in the Preadipocyte and Gpreadipocyte groups, respectively (Table 3). A total of 577 differentially methylated locations (DMLs) and 17 differentially methylated regions (DMRs) were identified and annotated, yielding a total of 152 DMGs (Fig. 5*A* and Table S8).

**Table 3:** Information about methylation

Sample ID	Methylated CpG	Total CpG	Methylated/total CpG (%)	Methylated CH	Total CH	Methylated/total CH (%)
Control 1	6,492,621	9,731,994	66.71	2,135,810	259,575,246	0.82
Control 2	3,546,862	5,684,476	62.40	1,491,918	162,976,295	0.92
Control 3	4,712,483	7,502,500	62.81	1,801,088	224,005,106	0.80
Treatment 1	3,066,181	4,861,055	63.08	1,214,285	146,354,723	0.83
Treatment 2	3,160,507	5,087,149	62.13	1,394,183	154,033,400	0.91
Treatment 3	3,272,467	5,171,611	63.28	1,096,083	138,180,171	0.79

Control: the Preadipocyte group; treatment: the Gpreadipocyte group.

Both GO and KEGG enrichment analyses were performed to investigate the potential functions of DMGs. The DMGs were mostly enriched in the GO terms of actin cytoskeleton, adherens junction, protein homodimerization activity, and signaling receptor binding, and KEGG pathways including phospholipase D signaling pathway, lysine degradation pathway, cell adhesion molecules, PI3K–Akt, and Jak–STAT signaling pathway (Fig. 5*B* and Table S9). These results confirmed that there are significant differences between the GM-preadipocytes and transdifferentiated GM-preadipocytes in both the transcriptome and DNA methylome levels. The function of DMGs was similar to that of DEGs, mainly focusing on cell cycle, cell adhesion, maintenance of cell architecture, and stability.

The gene expression was affected with DNA methylation by modulating bindings of upstream TFs. In this study, a total of 25 DMGs that were methylated in promoter region were screened, and these promoters were highly enriched in binding motifs of different TFs, including BTB domain and CNC homolog 2 (BACH2), CCCTC-binding factor (CTCF), and Spi-1 proto-oncogene (Fig. 5*C* and Table S10). The interactions of TF with their target genes were then used to build the TF-gene regulatory networks. We identified links between 14 TFs and 15 DMGs and constructed a putative regulatory network associated with GM-preadipocyte transdifferentiation (Fig. 5*D*).

### DNA methylation regulates GM-preadipocyte transdifferentiation by affecting gene expression

**Figure 6:**
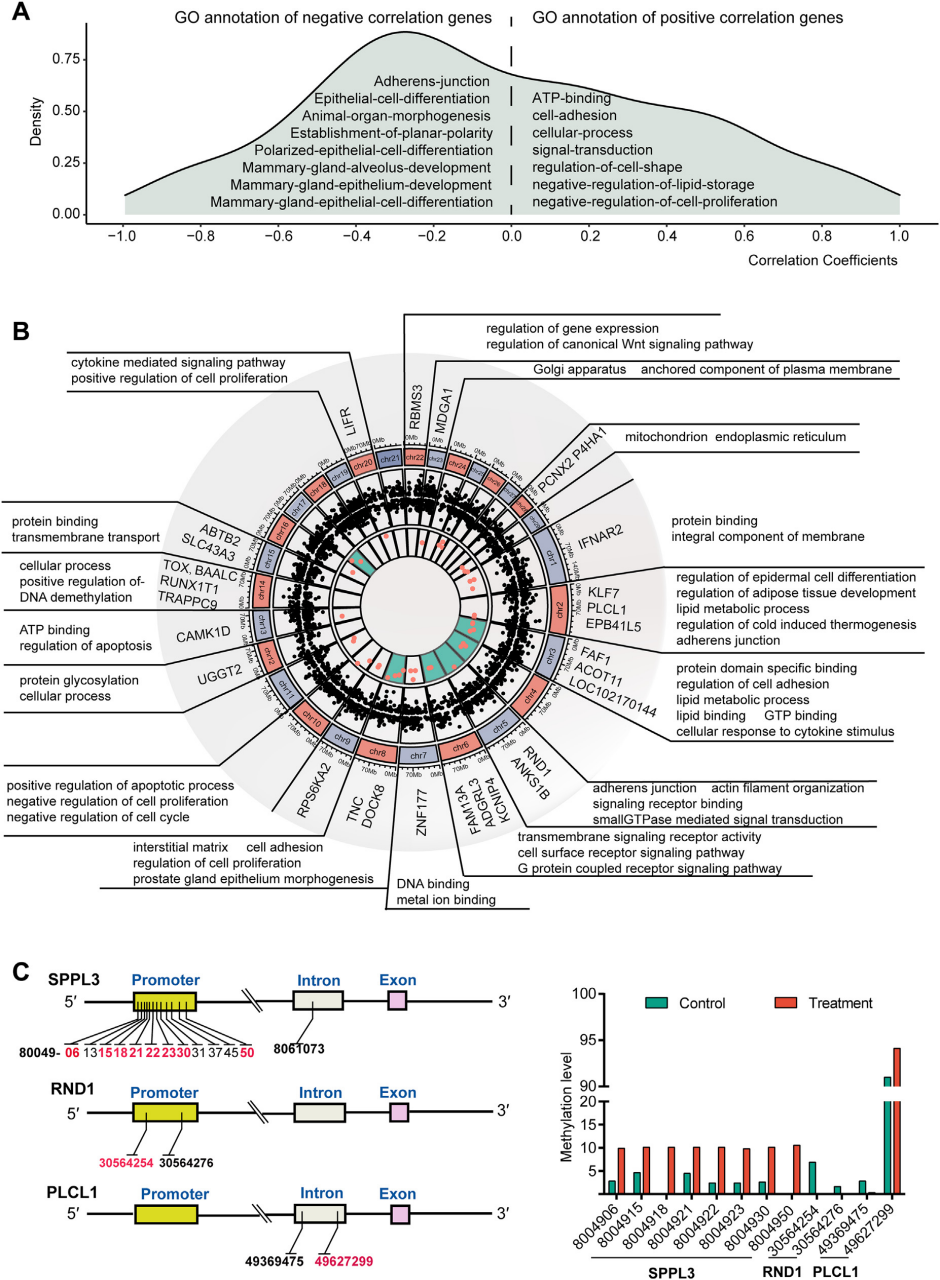
**DNA methylation regulates GM-preadipocyte transdifferentiation by affecting gene expression.***A*, the distribution of correlation coefficients between DNA methylation and the expression level of corresponding genes, and GO annotation of PGs or NGs. *B*, intersection of DEGs and DMGs, and their GO annotation. The *outer to inner circles* represent chromosome names, DEGs, and DMGs, respectively. *C*, the DNA methylation site distribution of three candidate DMGs. The differentially DNA methylated sites are indicated by *red number*. The *bar chart* represents the comparison of DNA methylation levels between two groups for each site. Control and treatment groups represent the Preadipocyte and Gpreadipocyte group, respectively. DEG, differentially expressed gene; DMG, differentially methylated gene; GM-preadipocyte, goat mammary preadipocyte; GO, Gene Ontology; NG, gene that has significantly negative correlation; PG, genes that have significantly positive correlation.

Pearson correlation coefficients were calculated to assess the relationship between gene expression and DNA methylation level of corresponding genes during the GM-preadipocyte-to-MEC transdifferentiation (Table S11). A total of 37 genes had significantly positive correlation (PGs), of which were related to cell adhesion, cell–cell junction, cell migration, and regulation of cell shape. We further found that 32 genes showed a significantly negative correlation (NGs) between DNA methylation level and gene expression, which were associated with planar polarity, regulation of cell adhesion, mammary gland epithelial cell differentiation, mammary gland epithelium development, and mammary gland alveolus development (Fig. 6*A* and Table S12). It seemed that function of NGs was closely related to the GM-preadipocyte-to-MEC transdifferentiation. Following that, through integration of the DEGs and DMGs, we found that 29 genes were methylated resulting in differential expression, and these genes were annotated into lipid metabolism, cell adhesion, and morphogenesis (Fig. 6*B* and Table S13).

Based on correlation analysis and integration of DEGs with DMGs, three DMGs were identified as being involved in methylation-mediated transdifferentiation: signal peptide peptidase like 3 (*SPPL3*), Rho family GTPase 1 (*RND1*), and phospholipase C like 1 (*PLCL1*). The DNA methylation site distribution of these three genes showed that differential methylation occurred at eight sites in the promoter of *SPPL3*, and the site of 8,004,918 location was most significant, whereas the site of 30,564,254 location had significant differential methylation in the promoter of *RND1*, and for promoter of *PLCL1*, the site of 49,627,299 location was also significantly differentially methylated (Fig. 6*C*). These differentially methylated sites may be important for epigenetic changes of GM-preadipocyte-to-MEC transdifferentiation.

### Construction of epigenetic regulatory networks

**Figure 7:**
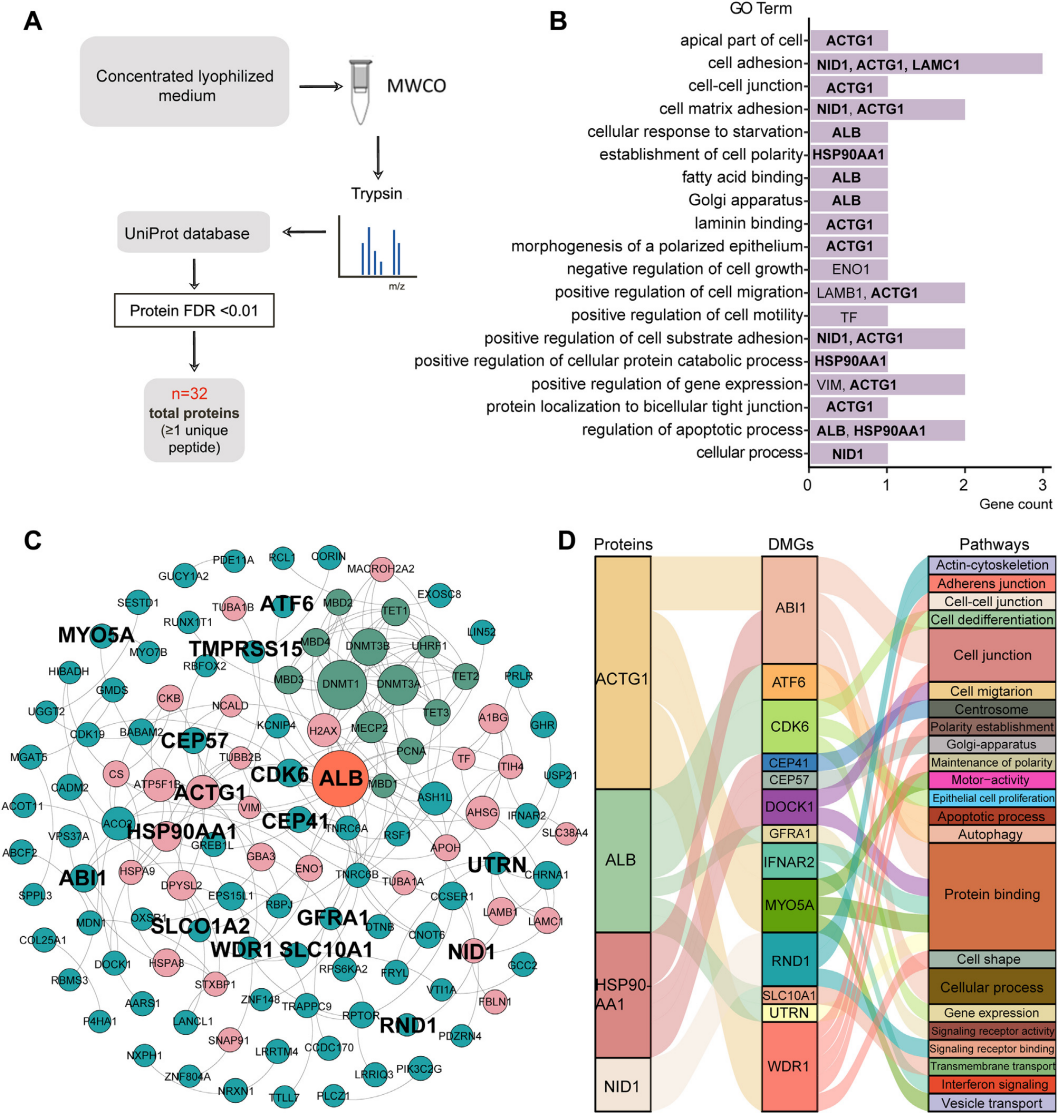
**The epigenetic regulation network for inducing GM-preadipocyte-to-MEC transdifferentiation.***A*, flowchart of secretory proteome detection. *B*, functional annotation of secretory protein in the CM. *C*, the construction of epigenetic regulation networks. The *green*, *blue*, and *pink circles* represent the DNA methylase, secretory protein of the CM, and DMGs, respectively. *D*, regulatory axis related to the GM-preadipocyte-to-MEC transdifferentiation. The connection in the *middle* is the corresponding relationship. CM, conditioned media; DMG, differentially methylated gene; GM, goat mammary; MEC, mammary epithelial cell.

The GM-preadipocytes proceed through epithelization, polarity establishment, and acinar development in the presence of CM. Protein mass spectrometry was used to detect proteins in CM to explore functional factors, and 32 proteins were identified (Fig. 7*A* and Table S14). Functions of these proteins were annotated into different GO terms (Fig. 7*B* and Table S15). Among the terms, the establishing cell polarity and morphogenesis of a polarized epithelium showed that secreted proteins could regulate the polarity of GM-preadipocytes. The cell adhesion, cell–cell junctions, and positively regulated cell substrate adhesion were associated with cell cycle and acinar formation. For identified proteins, albumin (ALB), nidogen 1 (NID1), actin gamma 1 (ACTG1), and heat shock protein 90 alpha family class A member 1 (HSP90AA1) were annotated into regulation of apoptosis process, cellular process, morphogenesis of a polarized epithelium, and establishment of cell polarity, respectively. Therefore, the results suggested that ALB, NID1, ACTG1, and HSP90AA1 are essential for regulation of different transdifferentiation processes.

The spectrum of transdifferentiation factors was then limited to paracrine mediators, which transfer signals from GMECs to GM-preadipocytes to induce transdifferentiation. An epigenetic regulatory network was constructed by integrating the DNA methylase, DMGs, and secretory proteins (Fig. 7*C* and Table S16). In the network, ALB was secreted from GMECs and impacted genomic methylation by interacting directly with DNA methylase and DMGs. In addition, ALB regulated epithelial cell proliferation, apoptosis, and autophagy by interacting with cyclin-dependent kinase 6 (*CDK6*) and activating TF 6 (*ATF6*), whereas ACTG1 regulated cell junction, cell shape, cell process, and cell activity by interacting with WD repeat domain 1 (*WDR1*), abl interactor 1 (*ABI1*), and myosin VA (*MYO5A*). HSP90AA1 regulated cell polarity establishment and cell junction by interacting with *ABI1*, and NID1 regulated cell adhesion by interacting with *RND1* (Fig. 7*D*).

Based on four kinds of analysis, including the correlation analysis, the overlap of DEGs and DMGs, TF regulatory network of DMGs, and epigenetic regulatory network, we found that TFs CTCF and BACH2 were the central regulators and involved in the transdifferentiation of GM-preadipocytes. For example, NID1 affected binding of CTCF to *RND1* by regulating the DNA methylation of *RND1* and ACTG1 affects the binding of BACH2 and *MYO5A* by mediating the DNA methylation of *MYO5A*.

## Discussion

The lack of polarized shape in secreted MECs may result in nonfunctional differentiation after disconnecting from the natural milieu. Tissue-specific genes can only be seen *in vitro* when extracellular matrix (ECM) signals can be received. In 3D culture, cell morphology, polarity, signal transduction, gene expression, and metabolism are similar to those of cells *in vivo*. The 3D cultured system provides an appropriate physiological condition for the study of complex cell–cell and cell–ECM interactions, which has been widely applied in various researches regarding mammary biology. In 3D culture condition, mouse MECs were used to study catheter invasion and elongation, morphogenetic procedures of alveolar genesis, and functional differentiation (19, 20), and supplement of key components induced milk synthesis of bovine MECs (21, 22). BME-UV1 (bovine MECs) formed acinar structures composed of polarized MECs when cells grew in Matrigel for 16 days (23). Many previous studies have indicated that Matrigel is an efficient 3D culture system for MECs of mouse, bovine, and human. In this study, the GMEC suspension was inoculated on Matrigel at day 0. During spontaneous induction of GMECs, we observed that cells were regularly and stably embedded in Matrigel at day 2, and the observation of cell morphology was continued every day after that in live cell imaging system. At day 4, cells rotated to form clusters and kept rotating and formed larger cell clusters at day 6. Up to day 8, many acinar structures could be observed, whereas we continuously cultured the cells for 14 days, morphology remained unchanged from day 8 to day 14. The GMEC morphology at these different time points in our results is similar to those of previous studies (24). Acinar structure efficiently formed from GMECs cultured in Matrigel *in vitro* was the experimental basis for the GM-preadipocyte-to-MEC transdifferentiation.

This study further provided a new understanding of adipocyte plasticity, revealing tissue's complexity. Browning is an example of adipose tissue adaptation in which brown adipocytes formed in a white adipose tissue (25, 26). The formation of fat pad is required for development of mammary epithelium, which presents another unique option of adipocyte plasticity. During the pregnancy and lactation periods, a substantial number of MECs with lactation function were grown in the mammary gland, out of which 30% were derived from ductal stem cells and 70% were from transdifferentiation of adipocytes, and indicate that mammary adipocytes are able to transdifferentiate into MECs with high expression of epithelial-specific protein (27, 28, 29, 30). Prior studies have demonstrated that preadipocytes labeled with PDGFRA transdifferentiated into MECs (5). In mammary gland, the interactions between cell–cell and cell–ECM provide a complex biochemical signal network, which is very important for the process of transdifferentiation (31, 32). To study the influence of human breast cell HBL-100 on adipose-derived stem cells (ASCs) in Matrigel, the expanded ASC tended to contract and shrank lasted for 72 h when treated with the CM made from HBL-100 culture medium; and ASCs formed acinar structures with high level of KRT18 in Matrigel (32). Similar to earlier findings, in our study, GM-preadipocytes expressed *PDGFRA*, *PDGFRA* (+) preadipocytes were induced by CM to transdifferentiate into MECs with high KRT18 expression, and subcellular structures with MEC features appeared. During transdifferentiation process of GM-preadipocytes, the GM-preadipocytes were cultured on Matrigel in CM for 14 days. The antennae of transdifferentiated GM-preadipocytes shrank at day 4, and the transdifferentiated GM-preadipocytes formed acinar-like structures at day 8, and there was no change from day 8 to day 14. The ultrastructural examination highlighted the existence of the adipose plasticity in adipocyte to MEC transdifferentiation (28). In line with the previous studies, our results achieved another adipose plasticity, which induced the GM-preadipocyte transdifferentiation into MECs.

Whether the process is reversible has not been further explored. For process opposite to the transdifferentiation of preadipocytes into MECs, a prior study has proved that mouse MECs could reversibly transdifferentiate into adipocytes during mammary gland involution (2); however, the molecular mechanism of MEC transdifferentiation *in vitro* has not been reported. The objectives of this work were to achieve the transdifferentiation of GM-preadipocytes into MECs with acinar features induced by the CM *in vitro* and identified potential candidate genes and regulatory networks that are related to the transdifferentiation, explaining why mammary adipose tissues disappear during pregnancy and lactation (33).

The cell–cell interactions and paracrine connections between MECs and stroma cells in mammary gland may reveal a possible mechanism of transdifferentiation (34, 35). Currently, the multiomics of GM-preadipocyte-to-MEC transdifferentiation was rarely examined, but we also compared the transcriptome and DNA methylome profiles between GM-preadipocytes and transdifferentiated GM-preadipocytes. The candidate genes related to the different regulatory processes of transdifferentiation were screened from DEGs and DMGs. Among these genes, DEGs and DMGs were mainly associated with immune response, cell adhesion, cell cycle, morphogenesis, and organization. In addition, phospholipase D signaling pathway and lysine degradation pathway were observed in enriched pathways of DMGs. Phospholipase D signaling pathway has participation in maintenance of cell structure and stability (36). While lysine degradation pathway includes two aspects: (i) lysine decomposition into the citric acid and (ii) carnitine. Carnitine has been shown to be involved in milk protein synthesis in previous studies (37, 38). On the other hand, detected TFs were enriched in promoters of DEGs and DMGs, including CTCF, BACH2, STAT1, Sp5 TF (SP5), and nuclear factor I B (NFIB). After cells were stimulated with the exogenous additive, they might respond to stimulation initially by interferon produced by STAT1-mediated immune response (39, 40). Whereas, BACH2 is a regulator of fatty acid profiles in mammary gland (41), CTCF regulates the localization of adhesin protein (42), and NFIB regulates the formation of alveolar epithelium *via* the Hippo signaling pathway (43, 44). These findings stated that the regulation of TFs is essential for GM-preadipocyte transdifferentiation.

The correlation between gene expression and DNA methylation levels was also calculated. The NGs were annotated into polarity establishment, regulation of MEC adhesion and differentiation, mammary gland epithelium development, and mammary gland alveolus development. Subsequently, three potential genes that epigenetically regulate transdifferentiation were screened when overlap of DEGs and DMGs was combined with the NGs. The *SPPL3* alters the pattern of cellular N-glycosylation by inducing the proteolytic release of glycosidase and glycosyltransferase extracellular domains (45), and the rate of glycan flux/the level of N-glycan biosynthetic enzyme determines the cellular behavior in reprogramming (46). Silencing *RND1* reduced MEC adhesion and polarity, whereas overexpression improved E-cadherin expression (47). The *PLCL1* could activate the browning of adipocyte plasticity (48). These reports illustrated that these candidate genes are crucial for GM-preadipocyte-to-MEC transdifferentiation. In addition, the secretory proteome in the CM was detected to construct complete regulatory networks. The epigenetic regulatory network was constructed by interacting DMGs and secretory proteins. In the regulatory network, ALB, ACTG1, NID1, and HSP90AA1 were deemed to be core proteins. Among these proteins, ALB involves in negative regulation of apoptotic process (49); ACTG1 regulates the cell adhesion, morphogenesis of a polarized epithelium, and the level of E-cadherin (50); NID1 promotes cell adhesion by connecting collagen IV and laminin (51); and HSP90AA1 participates in cell proliferation and apoptosis (52). In epigenetic regulatory axis, *RND1* was considered as a critical candidate gene regulated by secretory protein, which needs to be further verified in subsequent experiments.

Nevertheless, there are other limitations: the proteomes of GM-preadipocytes and transdifferentiated GM-preadipocytes were not detected, and the omics research is insufficient. Collectively in this study, three candidate genes and four candidate proteins were identified through integrated analysis of transcriptome, DNA methylome, and secretory proteome. These candidate functional factors and paracrine mediators play key regulatory roles in the whole processes of GM-preadipocyte-to-MEC transdifferentiation. This work provides new insights into the molecular mechanisms of adipocyte plasticity and mammary development during pregnancy and lactation.

## Experimental procedures

### Experimental design

**Figure 8:**
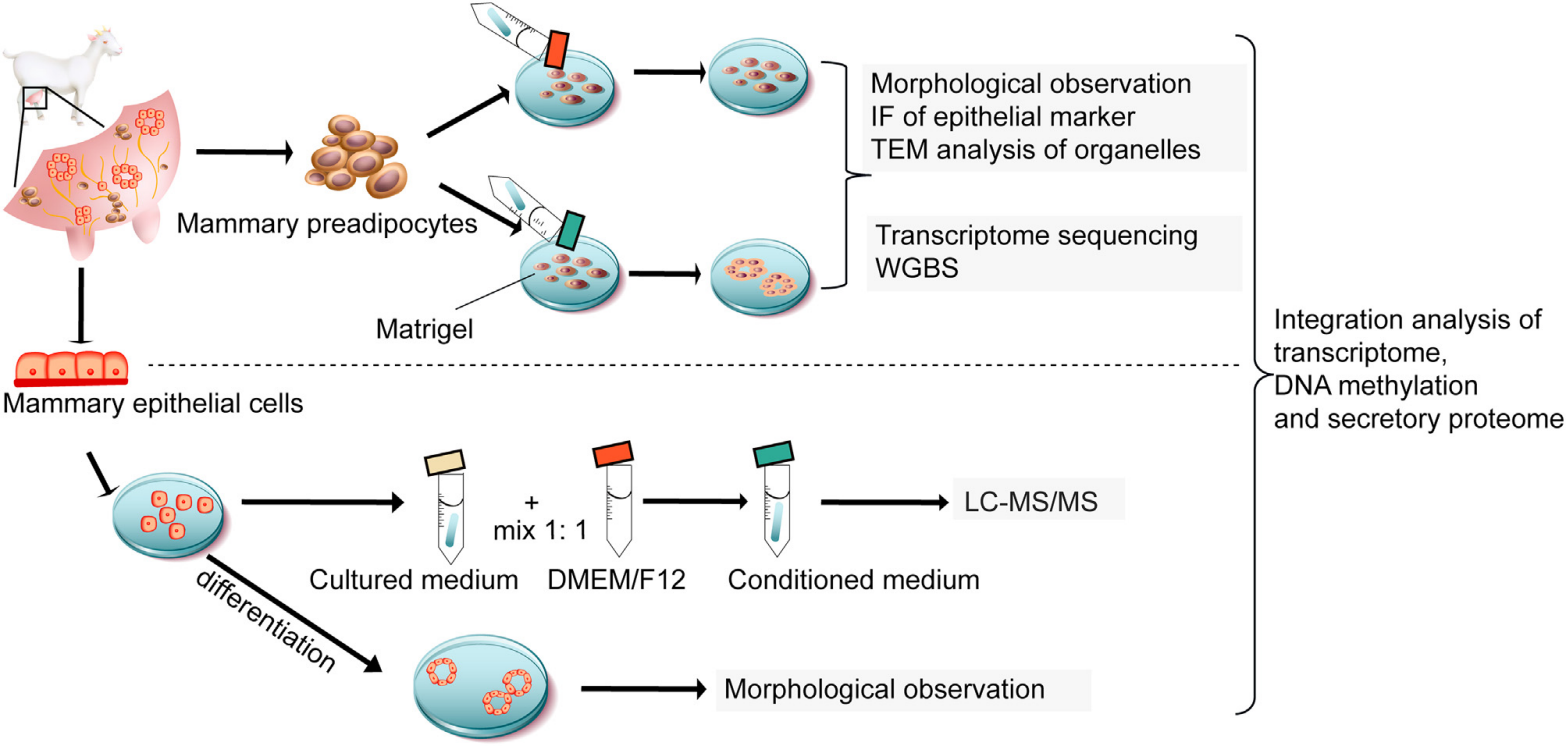
**Flowchart of experimental design**.

The GM-preadipocytes were isolated from adipose tissues around the goat mammary gland, whereas the GMECs were isolated from goat mammary gland. These cells were cultured in Matrigel (Corning), and the GMEC culture medium was collected as CM to induce the GM-preadipocyte transdifferentiation. Subsequently, GM-preadipocytes and transdifferentiated GM-preadipocytes were used for transcriptome sequencing and WGBS. Moreover, secretory proteins in CM were detected. The candidate genes, proteins, and epigenetic regulatory axes were screened through integration of transcriptome, DNA methylome, and secretory proteome (Fig. 8).

### Isolation, culture, and induced differentiation of GM-preadipocytes

The animal research ethics committee of Northwest A&F University approved the animal use protocol. GM-preadipocytes were isolated from adipose tissues around the goat mammary gland under sterile conditions. The adipose tissues were cut into 1 mm^3^ and digested at 37 °C with 2% (w/v) collagenase type I (Invitrogen) and then diluted with Dulbecco's modified Eagle's medium (DMEM)/F12 (Hyclone) for 2 to 3 h, and the cell suspension was centrifuged at 700*g* for 10 min. Then, pellets were resuspended in the growth medium containing 89% DMEM/F12, 10% fetal bovine serum (FBS; Gemini) and 1% penicillin–streptomycin (pen–strep; Solarbio). The undigested parts were filtered using 40-μm cell strainer, and harvested GM-preadipocytes were cultured in growth medium at 37 °C with 5% CO_2_.

Once cell confluence reached 90 to 100%, the growth-arrested cells were initiated to induce differentiation by treatment of the adipogenic induction medium containing 89% DMEM/F12, 10% FBS, 1% pen–strep, 1 μmol/l of dexamethasone (Solarbio), 250 μmol/l of 3-isobutyl-1-methylxanthine (Solarbio), and 5 μg/ml of insulin (Solarbio). After 2 days of adipogenic induction, cells were cultured in maintenance medium (89% DMEM/F12, 10% FBS, 1% pen–strep, and 5 μg/ml of insulin) for 8 days to maintain adipogenic induction, and maintenance medium was changed every 2 days.

### Isolation and culture of GMECs

GMECs were isolated from healthy peak-lactation goats as described previously (53). Briefly, mammary tissue was collected and rinsed repeatedly with D-Hank's (Hyclone) containing 3% pen–strep to remove adipose tissue and connective tissue. Parenchyma tissue was cut into 1 mm^3^ and placed in petri dishes pretreated with FBS and a few drops of DMEM/F12 growth medium consisting of 10% FBS, 1% pen–strep, 1% insulin, 0.1% epidermal growth factor (Invitrogen), and 2% hydrocortisone (Sigma). Near the tissue block, added a few drops of growth medium every 30 min to keep the tissue block wet. Two hours later, 3 ml of growth medium was added for tissue block culture, and GMECs were isolated and purified by differential adhesion. When cells were grown to 80 to 90% confluence, the medium was discarded and cells were treated with 0.25% trypsin–EDTA solution (Solarbio) for 5 min. Afterward, the collected cell suspension was centrifuged at 1000*g* for 4 min, and harvested cells were cultured in the growth medium at 37 °C with 5% CO_2_. The growth medium was changed every 2 days.

### Oil Red O staining

The Oil Red O staining was used to detect changes in lipid droplets in GM-preadipocytes at day 2, 5, and 8 of adipogenic induction. Briefly, cells were washed three times with PBS (Hyclone) and fixed with 4% paraformaldehyde (Solarbio) overnight at room temperature. Then, cells were dyed with working solution of Oil Red O (Solarbio), and dye was extracted by 60% isopropanol for 5 min at room temperature. The images were captured using an inverted microscope.

### Immunofluorescence

To verify some of the markers for GM-preadipocytes and GMECs at the protein level, we analyzed GM-preadipocytes markers (DLK1 and CD34) and GMEC marker (KRT18) by antibody-based immunofluorescence staining. The steps are as follows: at 90 to 100% cell confluence, then cells were washed three times with PBS containing 1% Tween-20 (Solarbio) and fixed with 4% paraformaldehyde. After 30 min, cells were treated with 0.1% Triton X-100 (Solarbio) and 5% bovine serum albumin (Solarbio) for 10 min, respectively. Afterward, cells were incubated with primary antibodies (Table S17) at 4 °C overnight on a low-speed shaker and then washed in PBS containing 1% Tween-20. After this step, FITC-conjugated secondary antibodies (Proteintech) were added, and cells were incubated for 1 h at room temperature. The nucleus was counterstained with 4,6-diamino-2-phenyl indole (Solarbio) for 5 min. Finally, the cells were visualized under the fluorescence microscope and confocal laser scanning microscope.

### Matrigel coating and cell culturing

The GM-preadipocytes and GMECs were cultured in Matrigel as described previously (16, 54). Matrigel was thawed in ice and diluted to 3 mg/ml with DMEM/F12. The bottoms of 48-well plate were coated with 250 μl Matrigel, and Matrigel-coated plates were placed at 37 °C for 1 h to solidify. Two types of cells were suspended using growth medium with 2% Matrigel (3D growth medium), inoculated into Matrigel-coated plates, and cultured in incubator at 37 °C with 5% CO_2_, respectively. The morphological changes were observed at the live cell imaging system. The medium was carefully changed every 2 days.

### Preparation of CM

During the process of CM preparation, GMECs were continuously cultured in Matrigel. The GMECs were cultured in Matrigel for 2 days and washed three times with preheated PBS. In the following 2-day culture, the 3D growth medium was replaced by serum-free 3D growth medium for culture medium collection. The resulting medium was centrifuged at 300*g* for 5 min, and then supernatant was collected and filtered with 0.22-μm filter. The filtered supernatant mixed with an equal volume of DMEM/F12 was subjected to a quality inspection, which included sterility testing and appearance inspection (32, 55). Finally, qualified medium was used as CM in induction of transdifferentiation and protein mass spectrometry.

### Induction of transdifferentiation

The transdifferentiation of GM-preadipocyte into MEC was carried out in Matrigel. The GM-preadipocytes were cultured for 24 h with serum-free 3D growth medium. After 24 h, cells were washed three times with preheated PBS, and the preheated CM was used to induce the GM-preadipocyte transdifferentiation (Gpreadipocyte group). Simultaneously, GM-preadipocytes were cultured with the serum-free 3D growth medium in Matrigel as the control group (Preadipocyte group). The medium was changed every 2 days, and induction of transdifferentiation was kept for 14 days. The morphological changes were observed every day using live cell imaging system.

### TEM

TEM was used to observe subcellular structures of GM-preadipocytes and transdifferentiated GM-preadipocytes as described previously (56). For cells of Preadipocyte and Gpreadipocyte groups, the medium was removed at day 8, and cells were washed three times with preheated PBS. Subsequently, the cells were fixed with 2.5% glutaraldehyde (0.1 M, pH = 7.2–7.4) for 5 h and washed three times with 0.1 M phosphoric acid buffer. Then, cells were fixed with 1% osmic acid (0.1 M, pH = 7.2–7.4) for 2 to 3 h and washed three times with 0.1 M phosphoric acid buffer for 15 min. Followed by twice gradient dehydration in a concentration series of ethanol (30, 50, 70, 80, 90, and 100% ethanol for 10 min, respectively). The samples were infiltrated with resin and embedded in paraffin using a capsule tool. Then, samples were fixed in an incubator at 55 °C for 48 h and cut into 50 to 70 nm-thick slices using an ultrathin slicer. Finally, samples were stained with 2% uranyl acetate and lead citrate for 30 and 15 min, respectively, and observed under TEM.

### Transcriptome data

#### Sample collection and RNA preparation

GM-preadipocytes and transdifferentiated GM-preadipocytes were collected to perform transcriptome sequencing. Cell recovery solution (Corning) was used to recover cells from Matrigel according to the manufacturer's instructions. The cell culture dishes were incubated with prechilled cell recovery solution at 4 °C for 20 min. After this step, the cells were separated from cell recovery solution by brief centrifugation (700*g* for 10 min). Finally, total RNA was extracted from collected cells using Trizol reagent (TaKaRa) according to the manufacturer’s protocol. The integrity of RNA was further detected by Agilent 2100 bioanalyzer (Agilent Technologies).

#### Complementary DNA library generation, sequencing, and mapping

The NEBNext Ultra RNA Library Prep Kit for Illumina (57) was used for complementary DNA (cDNA) library generation. The mRNA with ployA was enriched by oligo(dT) magnetic beads and further fragmented in NEB fragmentation buffer. First-strand cDNA was synthesized in M-MuLV reverse transcriptase system, and second-strand cDNA synthesis was subsequently performed using DNA polymerase I and RNase H. The 250 to 300 bp cDNA was screened for PCR amplification, PCR product was purified using AMPure XP beads, and preliminary quality of library was assessed on the Qubit 2.0 Fluorometer. Finally, the library was diluted to 1.5 ng/μl, and insert size of library was detected by Agilent 2100 bioanalyzer. The final libraries were subjected to sequence using Illumina NovaSeq 6000 (Illumina). The obtained raw reads were cleaned to remove adapter and low-quality reads using fastp (58) (version 0.19.7; the parameters: -q 5 -u 50 -n 15 -l 150). Then, HISAT2 (https://github.com/DaehwanKimLab/hisat2) (59) was used to map clean reads to goat reference genome ARS1. HISAT2 generated alignment was subjected to Subread featureCounts tool for generating a matrix of genewise counts.

#### Identification and functional enrichment analysis of DEGs

The DEGs were analyzed using the R package “DESeq2” (60), which is based on read counts for difference analysis. |Log_2_(foldchange)| ≥ 1 and *p* < 0.05 were used as the screening criteria. R package “piano” (61) was used to distinguish the function of downregulated and upregulated DEGs by gene set enrichment analysis, and the consensusScores function in “Piano” was used to estimate consensus gene set scores for each directionality class based on findings (gene set *p* values).

#### RT–qPCR assay and statistical analysis

Total RNA was reverse-transcribed into cDNA using PrimerScript RT Reagent Kit (Takara). The GAPDH was used as an internal control for gene expression analysis, and the primers used for RT–qPCR are listed in Table S18. The RT–qPCR was performed on the CFX Manager 3.1 analyzer system (Bio-Rad) using SYBR kit (Takara), and process was run as follows: 95 °C for 5 min, followed by 39 cycles of 95 °C for 30 s, 63 °C for 30 s, and 72 °C for 1 min. The expressions of genes were calculated using 2^−△△Cq^ method. The data of RT–qPCR were analyzed using unpaired *t* test in GraphPad Prism 6.0 software (GraphPad Software, Inc). For all analyses, *p* < 0.05 was considered statistically significant.

### WGBS data

#### Sample collection and DNA preparation

GM-preadipocytes and transdifferentiated GM-preadipocytes were collected for WGBS. The total DNA was extracted using HiPure Tissue DNA Micro Kit (Magnetec) according to the manufacturer’s instructions.

#### Library preparation, sequencing, mapping, and methylation calling

The DNA quality was detected using Qubit (Thermo), and the WGBS library was prepared as described previously (62). Covaris E220 focused-ultra sonicator was used for DNA fragmentation to produce DNA fragments ranging from 300 to 700 bp. Followed by a terminal repair and adenylation reaction, the cytosine methylation barcode was attached to DNA fragment. These DNA fragments were treated twice with bisulfite using EZ DNA Methylation-Gold Kit (Zymo Research). The Agilent Bioanalyzer 2100 system was then used to measure the insert size, and sequencing was carried out using DNBSEQ Platform (MGI Tech Co, Ltd). Finally, 100 bp paired-end reads were generated. The low-quality reads in raw data were filtered using fastp with parameter: -q 40 -u 20 -n 5 -l 30 -p 20 -w 4. BiSulfite Bolt (https://github.com/NuttyLogic/BSBolt) (63) was carried out to map clean reads to goat reference genome ARS1, and matrices containing methylation values and counts of methylated and total bases at each site were outputted for following analysis.

#### Identification of DMLs and DMRs

Based on methylation site information, the R package “Methylkit” (64) was used to analyze the DMLs and DMRs with 400-bp sliding window and 400-bp step. The DMR was defined as a region containing ≥4 DMLs. In addition, Methylkit was also used to annotate DMLs and DMRs obtaining DMGs.

#### TF-binding motif enrichment analysis

TF-binding motif enrichment analysis of promoter of DEGs and DMGs was carried out by MEME of JASPAR (65, 66). Promoters were defined as 5000-bp upstream and 5000-bp downstream from the transcriptional start site of each gene.

### Proteome data

#### Sample preparation

The collected CM for transdifferentiation induction was centrifuged with 200*g* for 10 min at 4 °C and then filtered with a 0.22-μm filter and added protease inhibitors (Roche). The resulting solution was dried by vacuum centrifugation, and it was redissolved with 1 ml of 50 mM NH_4_HCO_3_. For ultrafiltration, the Amicon Ultra-0.5 centrifugal filter device (Millipore) was pretreated according to the manufacturer's instructions. Then, the solution was concentrated using Amicon Ultra-0.5 centrifugal filters with 3 kDa cutoff (Merck Millipore). Filter-aided sample preparation was performed as described previously (67). Briefly, DTTs were added to the protein sample as reducing agent and then incubated at 37 °C for 1 h, and iodoacetamide was added as alkylating agent and reacted for 30 min at room temperature in the dark. After incubation, 0.5 μg of trypsin was added for an overnight digestion at 37 °C with shaking. The peptides were finally eluted and desalted on 100 μl C18 column (Thermo). Then, collected peptides were dried by vacuum centrifugation and redissolved with 10 μl 0.1% methanoic acid.

#### LC–MS/MS acquisition

The final solution was subjected to LC–MS/MS analysis using Orbitrap fusion mass spectrometer (Thermo). For LC–MS/MS analysis, using 0.1% methanoic acid and 0.1% methanoic acid, and 80% acetonitrile as mobile phase A and mobile phase B, respectively; The flow rate was adjusted to 300 nl/min; and analysis time, 0 min (8% phase B), 38 min (38% phase B), 43 min (48% phase B), 45 min (100% phase B), and 55 min (100% phase B). Default parameters were used for MS scan. We initially used Unified Protein Database (UniProt; https://www.uniprot.org/) to create a sample-specific database for identifying goat protein sequences. The Orbi RAW files were searched directly using Byonic (Protein Metrics) on Proteome Discoverer, version 2.2 software (Thermo), and spectra were searched by sample-specific database. The cleavage site of trypsin, the ion mass tolerance of 0.8 Da fragment, and the parent ion tolerance of 10.0 ppm were used as parameters. In addition, cysteine carbamidomethylation was defined as fixed and methionine oxidation and N-terminal acetylation as variable modifications. The proteome with high confidence and at least one unique peptide was collected for further analysis.

#### Functional enrichment analysis

DAVID (https://david.ncifcrf.gov/) was used to perform GO enrichment analysis, and KOBAS (http://kobas.cbi.pku.edu.cn/kobas3) was used for KEGG pathway functional enrichment analysis. These results were visualized using R package “ggplot2”.

## Data availability

All data are contained within the article, except the data deposited into a publicly accessible repository as follows:

The data were deposited in China National GeneBank (https://db.cngb.org/search/project/CNP0003568/ and https://db.cngb.org/search/project/CNP0003010/) embargoed until publication.

## Supporting information

This article contains supporting information.

## Conflict of interest

The authors declare that they have no conflicts of interest with the contents of this article.
