# Supplementary material for: In vitro transdifferentiated signatures of goat preadipocytes into mammary epithelial cells revealed by DNA methylation and transcriptome profiling
Source: J Biol Chem. 2022 Oct 17;298(12):102604. doi: 10.1016/j.jbc.2022.102604 (PMC9668736; doi:10.1016/j.jbc.2022.102604)
Supplement: Table S17 [file mmc17.docx]

**Table S17. Details of antibodies**

| Antiabodies | Source | Cotalog. | Dilution |
| --- | --- | --- | --- |
| DLK1 | Proteintech, Wuhan, China | 10636-1-AP | 1: 500 |
| CD34 | BOSTER, Wuhan, China | BA3414 | 1:500 |
| Cytokeratin 18 (KRT18) | Proteintech, Wuhan, China | 10830-1-AP | 1:500 |
