# Supplementary material for: In vitro transdifferentiated signatures of goat preadipocytes into mammary epithelial cells revealed by DNA methylation and transcriptome profiling
Source: J Biol Chem. 2022 Oct 17;298(12):102604. doi: 10.1016/j.jbc.2022.102604 (PMC9668736; doi:10.1016/j.jbc.2022.102604)
Supplement: Table S18 [file mmc18.docx]

**Table S18 Gene sequences of the primers used for RT-qPCR**

| Gene | Primer sequence (5ʹ–3ʹ) |
| --- | --- |
| *FABP4* | F: TTGCTACCAGGAAAGTGGCTG  R: AGCACCAGCTTATCATCCACAA |
| *PLCL1* | F: TCAGCGGACGTAGCAAACAT  R: TTCAGGGTCGGGTTGAGTTG |
| *MFGE8* | F: GCAGTTAGGAGAAAAGGTGAGGA  R: CTCACAGCCAAGGAGCTCAA |
| *Plin2* | F: TGCCAAAGGGGCTATGACTG  R: CCACGGACTTGGTCTTCTCC |
| *ACTG1* | F: AAATTGCCGCCCTCGTCATC  R: TAAGGGTTAGGATGCCACGC |
| *KLF11* | F: AGCCCAGACTCAAGAACAGC  R: AGGAATGGGGACGGAGATCA |
| *Bcl2* | F: GAGTTCGGAGGGGTCATGTG  R: GTTTTGATTTCCCAGCCTCCG |
| *Elf5* | F: CAGT TCTGC TGTGA CCAGTA  R: GAGTA ACCTT GTGAG CGGAT |
| *PPARγ* | F: CTTCACCACCGTTGACTTCT  R: TACAGGCTCCACTTTGATTG |
| *RND1* | F: GTGAC TCGGA CGCAG TATTA  R: TCCTC AGGTC GGTCT TACAA |
| GAPDH | F: CTTATGACCACTGTCCACGC  R: CCGTTGAGCTCAGGGATGAC |

F, forward; R, reverse.
