# Supplementary material for: In vitro transdifferentiated signatures of goat preadipocytes into mammary epithelial cells revealed by DNA methylation and transcriptome profiling
Source: J Biol Chem. 2022 Oct 17;298(12):102604. doi: 10.1016/j.jbc.2022.102604 (PMC9668736; doi:10.1016/j.jbc.2022.102604)
Supplement: Figure S1 [file mmc19.docx]

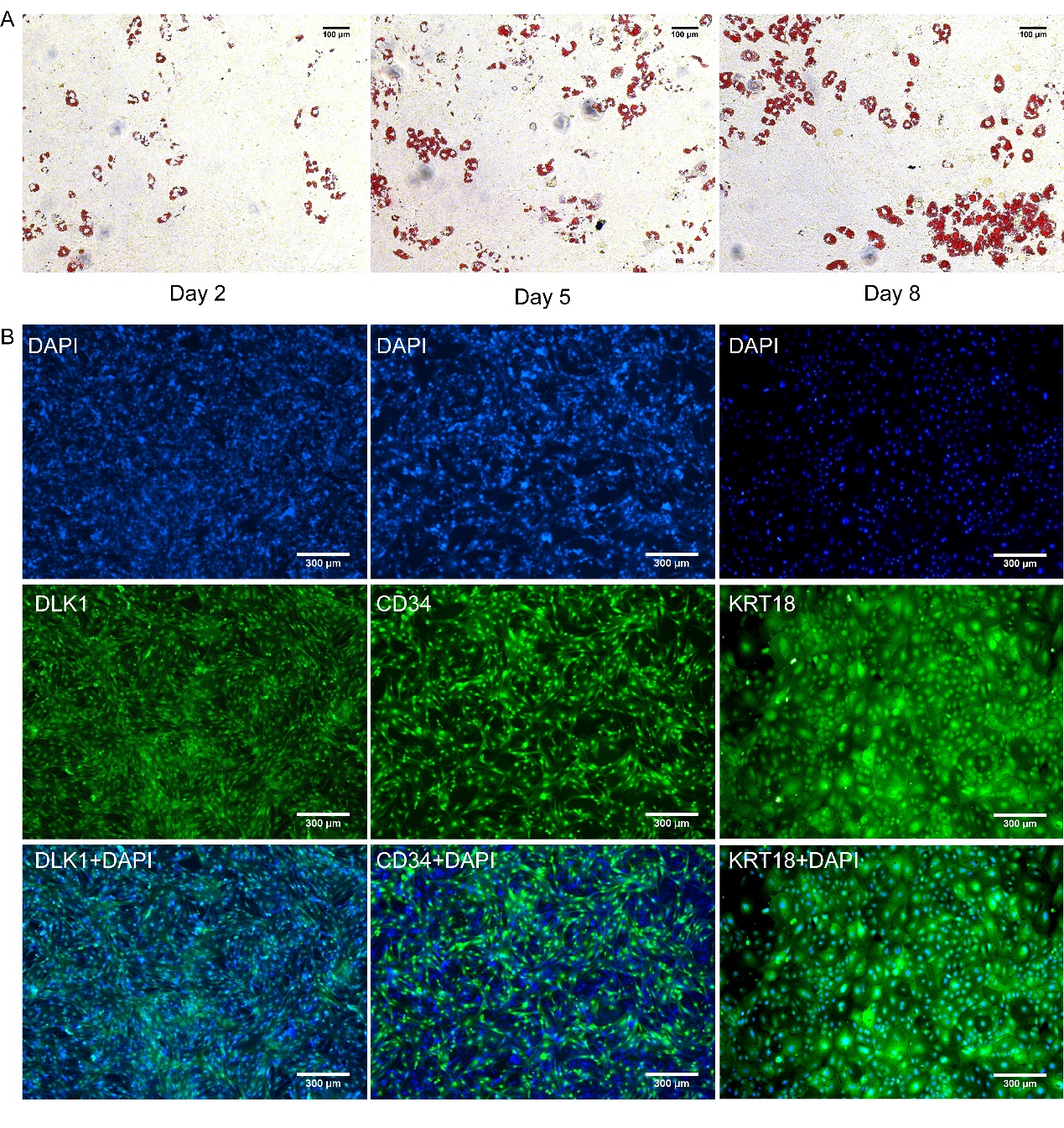


**Figure S1. Identification of GM-preadipocytes and GMECs.** A, oil red O lipid staining of GM-preadipocytes differentiated for 2, 5, 8 days. The growth medium of GM-preadipocytes was changed to adipogenic induction medium at day 0. The red part represents lipid droplets. B, immunofluorescence. Immunofluorescence of GM-preadipocytes using DLK1, CD34 monoclonal antibody (green), and DAPI (blue). Immunofluorescence of GMECs using KRT18 monoclonal antibody (green) and DAPI (blue).
