# Supplementary material for: In vitro transdifferentiated signatures of goat preadipocytes into mammary epithelial cells revealed by DNA methylation and transcriptome profiling
Source: J Biol Chem. 2022 Oct 17;298(12):102604. doi: 10.1016/j.jbc.2022.102604 (PMC9668736; doi:10.1016/j.jbc.2022.102604)
Supplement: Figure S2 [file mmc20.docx]

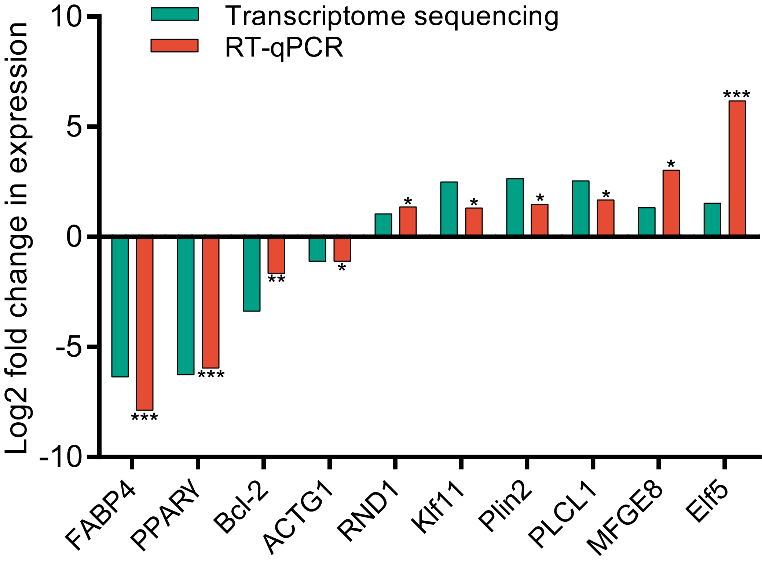


**Figure S2. Validation of selected DEGs through RT-qPCR.** The value is presented as the Log2 fold change between two groups. (* *P* < 0.05, ** *P* < 0.01, *** *P* < 0.001).
