# Supplementary material for: In vitro transdifferentiated signatures of goat preadipocytes into mammary epithelial cells revealed by DNA methylation and transcriptome profiling
Source: J Biol Chem. 2022 Oct 17;298(12):102604. doi: 10.1016/j.jbc.2022.102604 (PMC9668736; doi:10.1016/j.jbc.2022.102604)
Supplement: Figure S3 [file mmc21.docx]

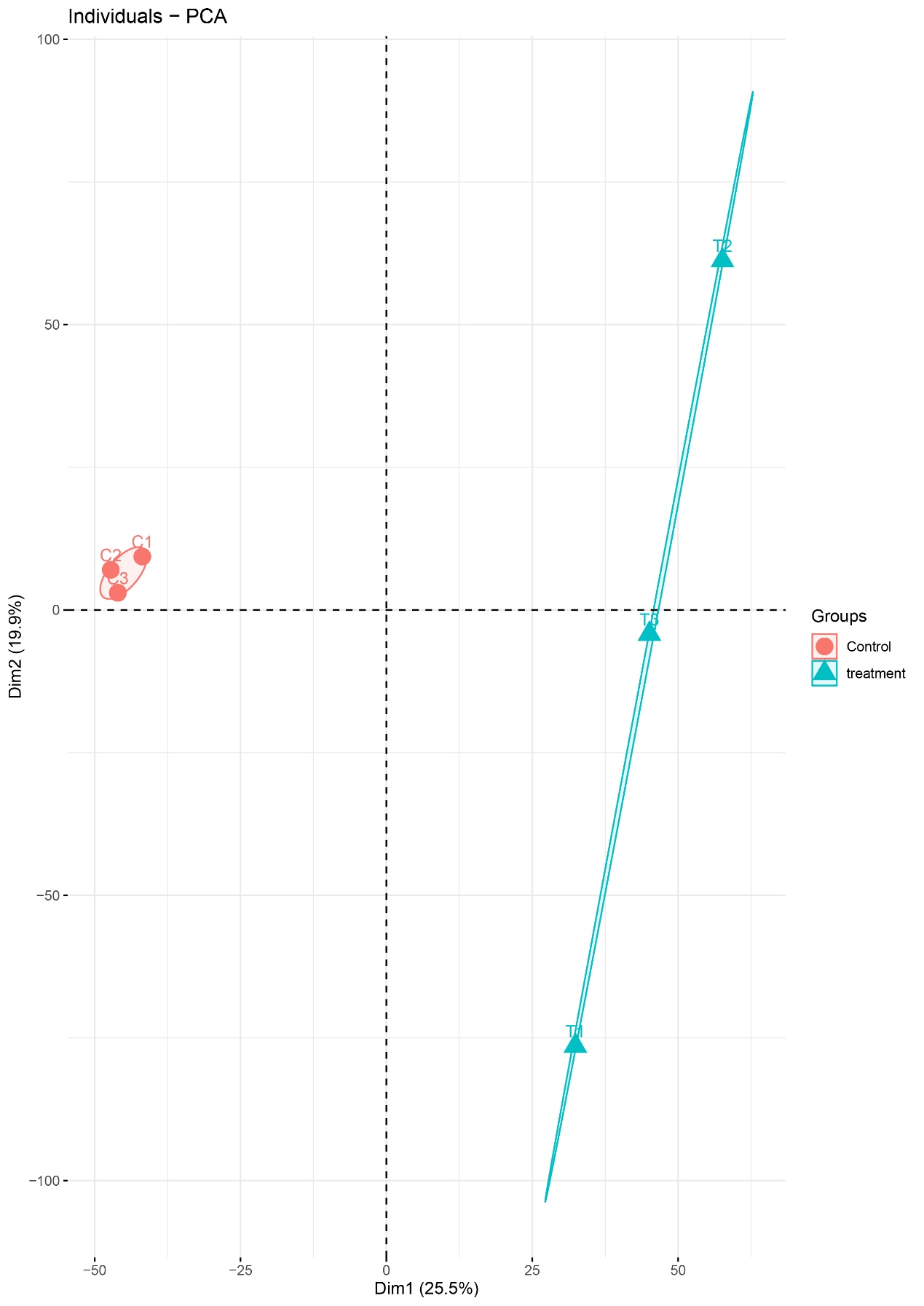


**Figure S3. PCA of GM-preadipocytes and transdifferentiated GM-preadipocytes for WGBS.** The Control group and Treatment group represent the Preadipocyte and Gpreadipocyte group, respectively.
